# 肺癌患者静脉血栓栓塞症的发生及其抗凝治疗

**DOI:** 10.3779/j.issn.1009-3419.2018.10.09

**Published:** 2018-10-20

**Authors:** 晖 杜, 军 陈

**Affiliations:** 300052 天津，天津医科大学总医院肺部肿瘤外科 Department of Lung Cancer Surgery, Tianjin Medical University General Hospital, Tianjin 300052, China

**Keywords:** 肺肿瘤, 静脉血栓栓塞症, 发生, 抗凝治疗, 低分子肝素, Lung neoplasms, Venous thromboembolism (VTE), Occurrence, Anticoagulant therapy, Low molecular weight heparin (LMWH)

## Abstract

目前，肺癌的发病率和死亡率在全世界范围内居首位。静脉血栓栓塞症（venous thromboembolism, VTE）是一种公认的肺癌并发症，也是肺癌患者的主要死因之一。癌症自身因素、患者自身因素以及治疗相关因素都是导致肺癌患者发生VTE的主要原因。肿瘤细胞可产生组织因子（tissue factor, TF）、癌性促凝物质（cancer procoagulant, CP）、炎症因子和细胞因子，从而直接激活凝血；其中TF过度表达是肿瘤患者血栓形成的主要机制之一。2016年美国胸外科医师协会（American College of Chest Physicians, ACCP）发布的第10版肿瘤患者VTE防治指南（AT-10）指出，抗凝治疗是肺癌患者合并VTE的基本治疗措施；其中低分子肝素（low molecular-weight-heparin, LWMH）被认为是首选的抗凝药物，但要注意出血风险。

## Abstract (en)

Lung cancer is the first leading cause of morbidity and mortality in the world. Venous thromboembolism (VTE) is a recognized complication in patients with lung cancer, which is one of the leading cause of death in lung cancer patients. The cancer-related, patient-related and treatment-related factors are the main causes of VTE in lung cancer patients. Malignant cells can directly activate blood coagulation by producing tissue factor (TF), cancer procoagulance (CP), inflammatory factors and cytokines; And the one of predominant mechanisms in cancer-related thrombosis is the overexpression of TF. The 10^th^ edition of the antithrombotic therapy guidelines for VTE with cancer patients (AT-10) published in 2016 by American College of Chest Physicians (APCC) recommended that anticoagulant therapy is the basic treatment for patients with lung cancer complicated with VTE; And low molecular-weight-heparin (LMWH) is preferred as an anticoagulant drug, but can be use with caution due to increasing risk of bleeding.

肺癌是目前全世界范围发病率和死亡率最高的恶性肿瘤，已成为当前研究的热点问题^[1]^。VTE包括肺栓塞（pulmonary thromboembolism, PE）和深静脉血栓（deep venous thrombosis, DVT），是一种公认的肺癌并发症，在肺癌患者中有极高的发病率及死亡率^[2, 3]^。据估计约有4%-20%的癌症患者经历过VTE^[4, 5]^。DVT主要发生在下肢，称为下肢深静脉血栓（lower extremity venous thrombosis, LEDVT），LEDVT包括下肢近端DVT和小腿DVT，小腿DVT又分为胫腓静脉血栓和小腿肌间静脉血栓（calf muscle venous thrombosis, CMVT）^[6, 7]^。

CMVT其发病隐匿，在临床多无症状，也很少引起临床医师和患者的重视，如果不给于治疗，部分CMVT会向近端进展，并可能导致肺栓塞^[8]^。因此本文给予一定介绍。CMVT是最常见的LEDVT之一，既可孤立出现，又可与下肢近端DVT或者胫腓静脉血栓并存^[8]^。相关研究提示：在出现DVT症状和体征的患者中，小腿肌间静脉被证明是血栓形成的最常见部位；在确诊为DVT的患者中，47%-79%的患者存在CMVT^[9-11]^。Macdonald等的研究了孤立性CMVT在未治疗患者中的传播情况，结果显示16.3%的CMVT延伸到邻近的胫腓静脉，或更高的水平，但只有2.9%的CMVT进展到了腘静脉水平。Macdonald等也证明90.9%的CMVT患者在发病2周内进展^[12]^。

肺癌患者的VTE事件会对其后续治疗产生严重后果，如出血风险、化疗延迟、血栓复发、生活质量的下降以及医疗资源的过度消耗等^[13, 14]^。有报道称，伴有VTE的恶性肿瘤更具有侵袭性，且预后更差^[15]^。伴有VTE发生的恶性肿瘤患者在6个月内死亡率增加了2倍多，1年内死亡率增加了3倍以上；VTE已成为癌症患者的第二大死因，也是癌症患者术后最常见的死因^[16]^。本文将对肺癌患者VTE发生的危险因素、发病机制、以及肺癌患者VTE的抗凝治疗进行讨论。

## 1 肺癌患者VTE发生的危险因素

Rudolph Virchow在1884首次提出，血栓发生的主要原因是：血管内皮损害、血流瘀滞和高凝状态^[17]^。这三种原因在临床肺癌患者中可归类为：癌症自身因素、患者自身因素以及治疗相关因素（表 1）。

**1:** 肺癌患者VTE发生的相关危险因素 Risk factors associated with VTE in patients with lung cancer

Lung cancer-related factors	Treatment-related factors	Patients-related factors
Histological type of lung cancer; Stages of lung cancer (Advanced lung cancer is an independent risk factor); Active lung cancer	Chemotherapy; Hormone therapy; Surgical treatment; PICC; Targeted therapy; Antiangiogenic therapy; EPO; Blood transfusion, *etc*.	Bed rest; History of VTE; Obesity; Trauma; Pregnancy; Leukocyte, Platelet elevation; Complications (Infection, Heart failure, *etc*.)

### 1.1 癌症自身因素

肺癌的组织学类型及分期与VTE的发生有着密切联系。NSCLC患者VTE的发病率高于SCLC患者；肺腺癌患者VTE的发病率高于肺鳞状细胞癌患者^[18]^。Tagalakis等^[19]^对493例NSCLC患者进行回顾性研究显示：13.6%的患者存在DVT；在伴有VTE的NSCLC患者中，腺癌占60%，而鳞状细胞癌占25%。一项对91, 933例肺癌患者的研究显示，腺癌患者2年内VTE的发病率为5%，鳞状细胞癌2.6%，大细胞癌3.2%，腺癌与鳞癌的差异有统计学意义（HR=1.9, 95%CI: 1.7-2.1）^[18]^。晚期肿瘤是VTE发生的独立高危因素^[20]^。VTE的出现与癌症的分期有明显的关系^[21]^。我科室对231例肺癌患者入院时行双下肢静脉彩超检查，结果提示12例患者存在LEDVT，且伴有远处转移的患者（包括肺癌N3淋巴结转移）更易发生LEDVT，差异具有统计学意义（*P* < 0.05）。

### 1.2 治疗相关因素

手术、化疗、经皮外周静脉插管（peripherally inserted central catheter, PICC）、靶向治疗等均增加了VTE的发生风险，但化疗与VTE发生的相关性更强^[22]^。在过去的二十年中，人们越来越多地认识到化疗的独立危险因素。接受化疗的癌症患者每年VTE的发病率估计为11%，这一风险可能会上升到20%或更高，这取决于所使用化疗药物的类型；同时，化疗使癌症患者VTE的复发风险增加了2倍^[4]^。相关文献报道：化疗诱导癌症患者发生VTE的发病机制可能是以下两种：（1）化疗对内皮细胞的直接毒性作用导致血管壁损伤，从而激活内皮细胞释放TF（外源性凝血级联反应的起始蛋白）；（2）化疗诱导细胞凋亡而释放促凝物质^[23]^。除了化疗外，许多化疗辅助治疗也增加了VTE的发生风险。但是，在临床上许多化疗患者是术后辅助化疗，这部分患者的VTE事件是由化疗引起还是由手术引起且术后一直存在，值得进一步研究。

手术治疗同样会增加肺癌患者VTE的发生风险。据估计，进行手术的肺癌患者与非手术的肺癌患者相比，术后DVT的发生风险增加2倍，PE的发生风险增加3倍^[2]^。相关资料提示：肺癌术后患者VTE的发病率约为7.4%，术后7天是VTE的发病高峰，其发生风险会持续到术后3个月^[24, 25]^。CMVT在肺癌术后更为常见，我科室对101例肺癌手术患者（术前双下肢彩超提示：均无下肢血栓发生）在术后1周内行双下肢静脉彩超检查，结果提示30例患者术后新发LEDVT。仅有1例患者是CMVT合并同侧胫腓骨静脉血栓，剩余均是CMVT，无胫腓静脉血栓或者是下肢近端DVT发生。手术引起肺癌患者发生VTE的原因包括：手术时的特定体位造成静脉血液瘀滞；手术造成大量的血管损伤而激活凝血纤溶系统；术后长期卧床；肺组织的减少等^[26]^。肿瘤的不完全切除、术后使用抗血管生成药物、靶向药物应用和术前D-Dimer水平升高等因素都会增加术后VTE的发生风险^[27]^。

### 1.3 患者自身因素

相关研究显示：恶性肿瘤患者VTE的总患病率为4%-20%，其中男性的发病率高于女性^[2]^。其他影响肺癌血栓形成的危险因素有：中心静脉导管、固定、口服避孕药、高龄、创伤、既往静脉血栓病史、妊娠、D二聚体水平升高、C反应蛋白升高、可溶性P-选择素升高、体重指数≥35 kg/m^2^、抗磷脂抗体升高、化疗前血小板计数超过350×10^9^/L或白细胞计数超过11×10^9^/L等^[2]^。

## 2 肺癌患者VTE发生的病理生理机制

肺癌患者VTE发生的病理生理机制是非常复杂的，这些机制主要包括凝血和纤溶系统的激活、炎症反应、急时相反应、细胞凋亡以及细胞因子的产生等^[28-30]^。恶性肿瘤细胞可产生TF、CP、细胞因子和炎症因子等，从而直接激活凝血^[31]^。相关研究^[32, 33]^表明，TF的过度表达是癌症相关VTE发生的主要因素。TF不仅能激活外源性凝血级联途径，还能刺激肿瘤血管生成^[34]^。TF调节血管生成的最常见机制是通过上调血管内皮生长因子（vascular endothelial growth factor, VEGF）和下调血小板反应素（thrombospondin, TSP）^[35]^。CP是一种68 KDa蛋白酶，能直接激活凝血因子X（coagulation factor X, FX），同时也能激活血小板^[28, 36, 37]^。

肿瘤细胞释放的细胞因子包括白细胞介素-1β（interleukin 1β, IL-1β）、肿瘤坏死因子-α（tumor necrosis factor-α, TNF-α）、VEGF等，这些细胞因子可以诱导血管内皮细胞产生TF，对凝血有重要作用^[4, 38, 39]^。肿瘤所致的炎症反应也能增加纤维蛋白原（fibrinogen, FIB）、凝血因子Ⅷ（coagulation factor Ⅷ, FⅧ）和血管性假血友病因子（von Willebrand factor, vWF）等急时相蛋白的产生，从而促进血栓形成^[31, 40]^。

肿瘤细胞促进VTE形成的另外一种重要机制是通过与内皮细胞、血小板、白细胞等接触粘附，从而激活局部凝血，促进血小板活化聚集和刺激白细胞释放细胞因子^[4, 28, 41]^，见图 1。

**1:**
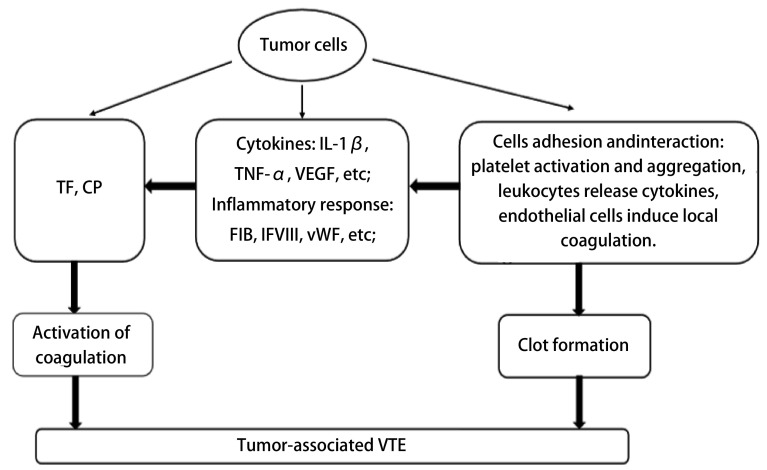
与肿瘤细胞相关的血栓形成机制 Mechanism of thrombogenesis associated with tumor cells

## 3 肺癌患者VTE的抗凝治疗

2016年美国胸外科医师协会（American College of Chest Physicians, ACCP）发布的第10版肿瘤患者VTE防治指南（AT-10）指出，有效的预防和治疗可以降低恶性肿瘤患者VTE的发病率及提高其生存率，并建议高风险的住院癌症患者应采取预防措施；接受大型手术的癌症患者术前或者术后尽早采取预防措施。低分子肝素（low molecular weight heparin, LMWH）被认为是预防和治疗癌症患者发生VTE的首先方案^[42]^。

AT-10防治指南依据循证医学的原则将证据级别分为3个等级：强度证据（A级）指无严重局限性的随机化研究；中度证据（B级）指存在严重局限性的随机化研究，如结果不一致或方法学有缺陷；低度证据（C级）指多来自于观察性研究。根据不同证据级别又给予不同的推荐等级（1：强力推荐；2：选择性推荐）。AT-10防治指南：将抗凝的时间段分为4周-6周，3个月，3周-12个月，延长治疗未设停药期限四种；并一直提倡根据初始抗凝后VTE再发风险来评估是否需要延长抗凝^[42]^。

AT-10防治指南建议：非卧床的癌症患者全身化疗期间，不推荐常规应用抗凝药物预防VTE（Grade 1C）。卧床的癌症患者存在发生VTE的中危风险，如无绝对禁忌证。推荐常规抗凝治疗（Grade 1A）^[42]^。在对1, 150例卧床癌症（279例肺癌）化疗患者进行预防VTE发生的临床试验时，那屈肝素或安慰剂在化疗期间被应用。结果显示，使用那屈肝素可以减少卧床癌症化疗患者VTE的发生率，但患者的出血相关并发症相应增加^[43]^。

美国临床肿瘤学会（American Society of Clinical Oncology, ASCO）2014版指南推荐：接受手术的癌症患者都应给予预防性抗凝治疗，抗凝治疗应在术前给予或术后尽早实施。建议常规使用LMWH抗凝，术后抗凝药物的应用至少持续7 d-10 d。术后有残留病灶、肥胖、既往有VTE病史的高危患者接受恶性肿瘤大手术时，抗凝治疗可延长至4周^[44]^。

CMVT发病隐匿，多见于外伤后或者手术后的急性发病，并且常孤立性存在。CMVT的治疗，目前尚缺乏全国性或者统一性的治疗指南，也无大型循症医学证据，根据血栓的病理特点，CMVT若不抗凝，则血栓会进一步蔓延为胫腓静脉血栓或者是下肢近端DVT，脱落后则可能引起肺栓塞；但若进一步抗凝治疗，则存在抗凝持续时间、出血等问题^[45-47]^。相关研究报道：154例CMVT患者均经血管彩超确诊，抗凝治疗时限1个月52例，3个月48例，6个月54例，均联合压力袜治疗。治疗后6个月随访彩超结果显示，3组患者治疗效果差异无统计学意义（*P* > 0.05）。全组无肺栓塞发生，出血事件5例，进一步发展为深静脉血栓2例；停止抗凝后6个月-18个月复发或发生深静脉血栓共14例^[48]^。

针对VTE的癌症患者，AT-10指南建议使用LMWH，优于使用维生素K拮抗剂（vitamin K antagonist, VKA）（2B级）或是达比加群、利伐沙班、阿哌沙班或依度沙班（2C级）。对于合并癌症的LEDVT或是PE患者，除非是高危出血风险，均推荐给予延长抗凝治疗（未设停药期限）^[42]^。对于复发性VTE的肺癌患者建议常规使用低分子肝素抗凝，如果之前接受VKA，抗凝应该换成LMWH，如果之前接受的是LMWH抗凝，则剂量应增加20%-25%^[49]^。

## 4 结论

目前有关对肺癌合并VTE的危险因素、发病机制以及抗凝治疗的研究已经很多，国内外也相继出台了一系列肿瘤相关性VTE防治的专家共识，但由于肺癌患者的VTE防治涉及多学科，循证医学资料多而复杂，缺少系统的归纳分析，同时鲜有来自国内的循证医学证据被指南采纳引用。因此，医务人员在进行临床实践时应谨慎、客观、严格地应用指南，对肺癌合并VTE的患者进行规范化、个体化诊疗。同时，也期待未来能有更高级别的循证医学证据，为肺癌VTE患者的临床诊疗带来更好的方案。
